# Occurrence and Exposure Assessment of Deoxynivalenol and Its Acetylated Derivatives from Grains and Grain Products in Zhejiang Province, China (2017–2020)

**DOI:** 10.3390/toxins14090586

**Published:** 2022-08-25

**Authors:** Yiming Chen, Ronghua Zhang, Enyu Tong, Pinggu Wu, Jiang Chen, Dong Zhao, Xiaodong Pan, Jikai Wang, Xiaoli Wu, Hexiang Zhang, Xiaojuan Qi, Yinyin Wu, Lei Fang, Biao Zhou

**Affiliations:** 1 Zhejiang Provincial Center for Disease Control and Prevention, Hangzhou 310051, China; 2 Department of Epidemiology and Health Statistics, School of Public Health, Faculty of Medicine, Hangzhou Normal University, Hangzhou 311121, China; 3 Department of Critical Care Medicine, Sir Run Run Shaw Hospital, Zhejiang University College of Medicine, Hangzhou 310020, China; 4 Key Laboratory of Microbial Technology and Bioinformatics of Zhejiang Province, Hangzhou 310020, China

**Keywords:** deoxynivalenol, occurrence, dietary exposure, risk assessment

## Abstract

Deoxynivalenol (DON) together with its acetylated derivatives cause detrimental effects on human health, and the purpose of this study was to assess the prevalence of DON and its acetylated derivatives from grains and grain products in Zhejiang province, China, and to assess the risk of DON and its acetylated derivatives due to multiple consumptions of grains and grain products among the Zhejiang population. Food samples numbering 713 were collected, and the LC-MS/MS method was used to determine the toxins. The levels of toxins from grains and grain products were relatively low: DON was the toxin at the highest levels. The food frequency questionnaire was used to collect food consumption data. The result of exposure assessments showed that the population was overall at low levels of toxin exposure. The probable mean group daily intake of toxins was 0.21 μg/kg bw/day, which was far from the group provisional maximum tolerable daily intake of 1 μg/kg bw/day, but 0.71% of participants were at high exposure levels. Rice and dried noodles (wheat-based food) were the main sources of toxin exposure, and reducing the consumption of rice and dried noodles while consuming more of other foods with lower levels of toxins is recommended.

## 1. Introduction

Deoxynivalenol (DON), a type B trichothecene mycotoxin produced by *Fusarium* species, is most often encountered as contaminants of cereal grains, oil seeds, and beans [1,2]. Previous animal studies have reported that DON can cause oxidative stress and intestinal tract injury and lead to acute poisoning symptoms, including an upset stomach, vomiting, anorexia, dizziness, headache, abdominal pain, and diarrhea [3,4,5,6,7]. In particular, DON with neurotoxicity can also damage the brain [6,7]. Moreover, it is also a potent inhibitor of protein synthesis and cell division [8,9]. 3-acetyldeoxynivalenol(3-ADON) and 15-acetyldeoxynivalenol(15-ADON) are the acetylated derivatives of DON, and their toxic effects are similar to DON [7,10]. 

DON is one of the most common trichothecene found in commodity grains; therefore, the greatest potential exists for it to occur in finished food products [11,12]. China is a large grain-produce country, and the total grain output in China was 600.45 million tons in 2020. As principal part of grain output, the output of rice, wheat, and maize were 130.94 million tons, 259.33 million tons, and 204.27 million tons, respectively [13]. However, as the primary staple foods of traditional Chinese diet, these grains and their products are easily contaminated by DON [14], which renders great risk potentials relative to the Chinese population. Previous population-based studies showed that there was a food poisoning case at a school in Zhuhai city, Guangdong province, China, in 2019 [15] caused by DON contamination in commercial raw noodles, and many students began vomiting after eating breakfast. Moreover, a study showed that DON contamination in wheat flour may be involved in fluctuating high prevalence rates of Kashin–Beck disease (KBD) in children in the KBD areas in Qinghai Province, China [16], indicating the harmfulness of this toxin.

There have been several detoxification strategies applied to reduce DON contamination in foods, which can be classified into physical, chemical, and biological detoxification. Physical detoxification strategies usually utilize high-temperature heating, irradiation, or sorbents to degrade DON, which could cause a reduction in nutrients in food [17,18,19]. Chemical detoxification comprises using chemicals to degrade DON in food, but some compounds might leave behind toxic metabolites in foods, which might be risky [20]. Biological detoxification comprises using microorganisms to transform DON into substances that are less toxic than DON [21,22]. However, biological detoxification cannot make all converted products nontoxic, and some converted products are still toxic or even more toxic [23]. Although these detoxification strategies have certain effects, they may have many adverse effects on the food. Thus, it is essential to monitor DON in foods and to assess the risk of DON relative to consumers that are exposed. 

To monitor DON and assess the exposure risk quantificationally, many countries and organizations have set maximum levels (MLs) for DON in grains and grain products. In China, the ML for DON in grain and grain products is 1000 μg/kg [24]. The MLs for DON regulated by the European Union (EU) are 1250 μg/kg, 1750 μg/kg, and 1250 μg/kg in unprocessed wheat, maize, and rice, respectively, and are 750 μg/kg in most cereals intended for direct human consumption [25]. Moreover, Joint FAO/WHO Expert Committee on Food Additives (JECFA) has established a group provisional maximum tolerable daily intake (PMTDI) of 1 μg/kg bw/day for the sum of DON and its derivatives [26]. 

There have been many studies assessing the risk of population exposed to DON and its derivatives from grains and grain products worldwide [9,27,28,29,30,31]. In China, there also have been some related studies [32,33,34,35,36]. Most studies have showed that the exposure levels of DON and its derivatives of participants were acceptable, but some of the participants were at high exposure levels. However, there were few relative studies performed in Zhejiang, a province in the southeast of China. Thus, the objective of this study is to monitor DON and its derivatives from grains and grain products in Zhejiang and to assess the risks of these toxins to which Zhejiang residents were exposed. In this study, the food frequency questionnaire (FFQ) was used to collect food consumption data, and liquid chromatography coupled with the tandem mass spectrometry method was used to assess the prevalence of these toxins from grains and grain products. 

## 2. Results

### 2.1. Occurrence of DON, 3-ADON, and 15-ADON

The total number of food samples collected in Zhejiang province, China, was 713, and the prevalence of DON, 3-ADON, and 15-ADON in samples is showed in Table 1. 

DON was detected in 45.16%, 7.94%, 96.96%, 95.26%, and 30.65% of rice, millet, dried noodles, instant noodles, and maize grains, respectively, and the P50 levels of DON were 2.50 μg/kg, 2.50 μg/kg, 138.00 μg/kg, 136.00 μg/kg, and 2.50 μg/kg, respectively. According to No 1881/2006, 0%, 0%, 10.27%, 7.76%, and 0% of rice, millet, dried noodles, instant noodles, and maize grains comprise excessive concentrations of DON contamination, respectively. According to GB 2761-2017, 0%, 0%, 6.08%, 5.17%, and 0% of rice, millet, dried noodles, instant noodles, and maize grains comprise excessive concentrations of DON contamination, respectively.

3-ADON was detected in 11.83%, 0.00 %, 3.91%, 3.45%, and 1.61% of rice, millet, dried noodles, instant noodles, and maize grains, respectively, and the P50 levels of 3-ADON were 3.25 μg/kg, 3.75 μg/kg, 5.00 μg/kg, 5.00 μg/kg, and 3.75 μg/kg, respectively. 15-ADON was detected in 2.15%, 0.00%, 0.78%, 0.86%, and 24.19% of rice, millet, dried noodles, instant noodles, and maize grains, respectively, and the P50 levels of 15-ADON were 3.75 μg/kg, 3.75 μg/kg, 5.00 μg/kg, 5.00 μg/kg, and 3.75 μg/kg, respectively.

Overall, it showed that at least one of toxins were detected in all five foodstuffs. Among toxins, DON was the toxin at the highest levels in grains and grain products. Among foodstuffs, wheat-based food products (dried noodles and instant noodles) were had the highest levels of toxins.

### 2.2. Daily Consumption of Grains and Grain Products of Participants

Table 2 showed the daily consumption of grains and grain products of participants per kilogram of body weight. The P50 daily consumption of rice, millet, dried noodles, instant noodles, and maize grains of participants per kilogram of body weight was 3.67 g/kg bw/day, 0.00 g/kg bw/day, 0.10 g/kg bw/day, 0.00 g/kg bw/day, and 0.05 g/kg bw/day, respectively. A Wilcoxon rank sum test showed that the consumption of rice, dried noodles, instant noodles, and maize grains of children (age = 0–17) per kilogram of body weight was all significantly higher (*p* < 0.001) than adults (age = 18–59) and elderly (age ≥ 60), and the consumption of rice and dried noodles of children was much higher than that of adults and elderly in particular.

### 2.3. PDI of DON, 3-ADON, and 15-ADON of Participants

Table 3 showed the PDI of DON, 3-ADON, and 15-ADON of participants. For foodstuffs, rice and dried noodles were the main sources of toxin exposure. For toxins, the PDI of DON of participants was higher than that of 3-ADON or 15-ADON. 

### 2.4. Group PDI of DON and Its Derivatives That Participants of Different Ages Were Exposed to

Table 4 showed the group PDI of DON and its derivatives that participants of different ages were exposed to, and the mean group PDI was 0.21 μg/kg bw/day (<1 μg/kg bw/day), which indicated that participants were at low exposure levels in general. For children, adults, and elderly, the mean group’s PDIs were 0.31 μg/kg bw/day, 0.18 μg/kg bw/day, and 0.19 μg/kg bw/day, respectively. The Wilcoxon rank sum test showed that the group PDI of children was significantly higher than group PDI of adults (*Z* = −21.91, *p* < 0.001) and elderly (*Z* = −15.61, *p* < 0.001), while there was no significant difference between the group PDI of adults and elderly (*Z* = −0.02, *p* = 0.99). Among 6413 participants, 0.71% (46/6413) participants were at high exposure levels, and 2.14% of (32/1494) children, 0.27% (11/4002) of adults, and 0.33% (3/917) of elderly were at high exposure levels. The chi-square test showed that the rate of children at high exposure levels was significantly higher than that of adults (*χ*^2^ = 48.85, *p* < 0.001) and elderly (*χ*^2^ = 13.08, *p* < 0.001), while there was no significant difference between adults and elderly (*χ*^2^ = 0.07, *p* = 0.79). According to the contributions of different foodstuffs to toxin exposure, rice and dried noodles were the main sources of toxin exposure.

## 3. Discussion

This study investigated the prevalence of DON and its derivatives from grains and grain products in Zhejiang Province and assessed the risks relative to toxins that participants were exposed to. Overall, the results showed that the levels of DON and its derivatives from grains and grain products were relatively low, and Zhejiang residents were at low toxin exposure levels. A few of the residents had high exposure levels relative to the toxins.

The detection rates and the levels of DON contamination were relatively high in wheat-based foods, and the reason might be that wheat was more easily damaged by Fusarium head blight (FHB, mainly caused by *Fusarium graminearum*), which would cause high DON levels in wheat [37,38]. Compared with other subtropical provinces in China such as Jiangsu [39] (84.6%, 1407 μg/kg), Hubei [39] (100%, 6314.9 μg/kg), and Sichuan [40] (78.6%, 522.0 μg/kg), the wheat-based foods in Zhejiang Province (95.26–96.96%, 264.72–306.37 μg/kg) were at the lowest levels of DON. In other subtropical regions, a study in Southern Brazil [9] revealed that 77.9% of the wheat flour samples were contaminated by DON with a mean level of 234.17 μg/kg. Mercedes and their colleagues [41] evaluated the prevalence of DON in wheat flour in Argentina, and the result showed that 91.2% of samples were contaminated by DON with a mean level of 243 µg/kg. These studies showed that warm and humid conditions in subtropical regions favor *Fusarium* species’ growth and the production of DON [42,43].

Among the food samples, some of the wheat-based food (dried noodles and instant noodles) samples were in excessive concentrations of DON contamination. For dried noodles, the over-standard rates according to No 1881/2006 and GB 2761-2017 regulation were 10.27% and 6.08%, respectively. For instant noodles, the over-standard rates were 7.76% and 5.17% correspondingly. In other subtropical regions in China, such as Yunnan province, a study showed that the over-standard rate of DON in noodles was 0% [44]. The over-standard rates of DON were 7.89% and 1.30% in Jiangsu province [45] and Shanghai [46], respectively, in wheat and wheat-based foods according to GB 2761-2017. In other countries, a study showed that 1.10% of the wheat samples were in excessive concentrations of DON contamination in Poland [47] according to No 1881/2006, and 5.22% of wheat flour samples were in excessive concentrations in Hungary [48]. In Southern Brazil, 16% of the wheat flour samples were outside the limit when compared with the value of 750 μg/kg [49]. This is similar to other regions where some of wheat-based foods had exorbitant concentrations of DON contamination, and similar results were reaffirmed in this study, which requires high awareness. 

The mean group PDI of DON and its derivatives of the participants was 0.21 μg/kg bw/day, and 0.71% of participants were at high exposure levels. In previous studies in China, Yin and their colleagues [46] assessed the risk to DON, 3-ADON, and 15-ADON from wheat flour of exposed Shanghai residents, and the result showed that the group PDI of DON, 3-ADON, and 15-ADON was 0.40 μg/kg bw/day; 10.01% of participants were at high exposure levels. In Anhui province, the mean PDI of DON in grain and grain products of participants was 2.6 μg/kg bw/day, and 93.8% of participants were at high exposure levels [50]. In Shandong province, the mean PDI of DON in wheat-based foods of participants was 0.58 μg/kg bw/day, and 16.5% of participants were at high exposure levels [51]. Another study showed that the mean PDI of DON in maize and maize products of participants in Shandong was 0.02 μg/kg bw/day [33]. In other countries, the mean PDI of DON from maize-based foods of infants in Tanzania was 1.9 μg/kg bw/day, and 29% of infants were at high exposure levels [29] according to the PMTDI of 1 μg/kg bw/day set by JECFA. Gilbert-Sandoval and their colleagues assessed the risk of exposure to DON from Mexican maize and found that 8.6% of males and 4.2% of females were at high exposure levels [31]. Compared with population in those areas, Zhejiang residents were at relatively lower exposure risks.

Rice and dried noodles were the main sources for DON and the exposure of its derivatives. The reason might be that the consumption of rice and dried noodles was relatively high, and toxin levels of these foods were also relatively high. Rice was the primary staple food of Zhejiang population [52]. For dried noodles, as mentioned above, wheat was easily damaged by FHB, which might causehigh levels of toxins. In addition, it showed that the PDI of DON was higher than that of 3-ADON or 15-ADON.The reason might be that compared with 3-ADON and 15-ADON, DON was at the higher levels in grains and grain products. 

The group PDI of children was significantly higher than the group PDI of adults and elderly, and other studies also showed similar results. For example, Sirot and their colleagues [27] assessed the risk of exposure to DON and its derivatives from several types of foods, and the result showed that mean group PDIs of children and adults were 0.54–0.62 μg/kg bw/day and 0.37–0.41 μg/kg bw/day, respectively, and 5–10% of children and 0.5–0.7% of adults were at high exposure levels. In Brazil, the PDI of DON from wheat flour-based food products of population was assessed in a study, and the result showed that the PDI of DON of children, adults, and elderly was 1.20–1.28 μg/kg bw/day, 0.78–0.92 μg/kg bw/day, and 0.65–0.73 μg/kg bw/day, respectively. The reason might be that children were in a crucial period for physical development, and their daily food consumption per kilogram of body weight was relatively high. Indeed, the consumption of rice, dried noodles, instant noodles, and maize grains of children per kilogram of body weight was all significantly higher than that of adults or elderly in this study.

Although extensive efforts have been made towards evaluating the most important top five grains and grain products in Zhejiang, other grains and grain products, such as oats, breads, and cookies, were not investigated. Hence, the actual risk of exposure to DON and its derivatives from grains and grain products of Zhejiang population might be higher than reported herein.

## 4. Conclusions

Taken together, the levels of DON and its derivatives from grains and grain products were relatively low, and some of the samples were at excessive DON levels, indicating a potential risk to public health. Because of the high levels of DON contamination in wheat-based foods, monitoring toxins from wheat-based foods was particularly important. Moreover, the exposure levels of DON and its derivatives from grains and grain products of Zhejiang residents were also low in general. A few residents were at high exposure levels. Compared with adults and elderly, children were at higher exposure levels. To reduce the risk of exposure to DON and its derivatives, it is essential to develop novel detoxication methods to reduce their contamination in rice and wheat-based foods and consume other foods at lower levels of toxins.

## 5. Materials and Methods

### 5.1. Food Sample Collection

The sample size of foods was determined using the following formula [53]:

(1) $$N=\frac{Z^{2}\times \left[P\times \left(1-P\right)\right]}{d^{2}}$$

where *N* = sample size; *Z* = 1.96 for 95% confidence level; *p* = 0.5 for expected percentage of samples containing toxins; and *d* = 10% representing precision. According to the formula, a minimum of 96.04 samples had to be collected. In this study, 713 food samples for direct consumption comprised rice (n = 93; dry matter content: 86.7% [54]), millet (n = 63; dry matter content: 88.4% [54]), dried noodles (n = 263; wheat-based food; dry matter content: 88.5% [54]), instant noodles (n = 232; wheat-based food; dry matter content: 96.4% [55]), and maize grains (n = 62; dry matter content: 28.7% [54]), and these were collected randomly from different regions of Zhejiang province during the period of 2017–2020; detailed information of representative products from 2017 to 2020 is shown in Table S1. Food samples were collected from small-scale farms, retail shops, supermarkets, and online stores in duplicate (approximately 1.0 kg) by trained investigators. Collected samples were transported to the laboratory in a cooler with dry ice and kept frozen at −20 °C until chemical analysis. Food samples were cryopreserved at −20 °C and transported to the laboratory until further analysis.

### 5.2. Preparation of the Standard Solution

All investigators were trained for standard solution and sample analysis. Pure mycotoxins of DON, 3-ADON, 15-ADON and the isotope internal standard solutions of ^13^C_15_-DON (25 µg/mL), ^13^C_15_-3-ADON (25 µg/mL), and ^13^C_15_-15-ADON (10 µg/mL) were obtained from Sigma-Aldrich (St Louis, MO, USA). DON, 3-ADON, and 15-ADON measuring 100 μg were dissolved into 1 mL of pure acetonitrile to obtain a single standard stock solution at a concentration of 100 μg/mL. The three stock solutions were then mixed and diluted with pure acetonitrile to obtain a mixed standard stock solution at a concentration of 50 μg/mL for every mycotoxin, and three-isotope internal standard solutions were also mixed and diluted with pure acetonitrile to obtain a mixed isotope internal standard solution at a concentration of 1.25 μg/mL for every mycotoxin. All solutions were stored in darkness at −20 °C for further chemical analysis. 

### 5.3. Sample Analysis

Toxin extraction and measurements were performed according to National Manual for Risk Monitoring of Food Pollutants and Harmful Factors issued by the China National Center for Food Safety Risk Assessment [56]. In brief, every food sample (approximately 1.0 kg) was crushed into particles with a size less than 0.5–1 mm, and they were split into 0.1 kg. Then, the mixture of a 5.0 g plit sample and 20 mL of acetonitrile–water–formic acid (70/29/1, *v*/*v*/*v*) solution was thoroughly vortexed and centrifuged, and a 0.22 μm filter membrane was used to filtrate the supernatant. After that, the filtrate combined with a mixed isotope internal standard solution was ready for injection. An 8060-LC-MS/MS equipped with an electrospray ionization (ESI) source (Shimadzu, Japan) was used for sample analysis. Chromatographic separations of mycotoxins were performed on a BEH C18 column (2.1 × 150 mm I.D., 1.7 μm, Waters, Milford, MA, USA) and at a flow rate of 0.30 mL/min at 40 °C. The injection volume was 10 μL. Mobile phases A and B were water and acetonitrile, respectively. The linear gradient elution program was applied as follows: 0.0 min 5% B, 1.0 min 5% B, 1.5 min 20% B, 5.0 min 25% B, 5.5 min 100% B, 8.2 min 100% B, 8.5 min 5% B, and 12.0 min 20% B. The system was equilibrated for 2.2 min before the next injection, providing a total run time of 17 min. 

### 5.4. Method Validation and Quality Control

Method validation and quality control were also performed according the National Manual for Risk Monitoring of Food Pollutants and Harmful Factors [56]. In brief, the mixed standard stock solutions ranged from 5.0 ng/mL to 100.0 ng/mL with six concentration levels combined with mixed isotope internal standard solution to prepare the standard curve. To determine the limit of detection (LOD), six replicates were analyzed at each spiked concentration level until signal-to-noise ratios of 3/1 and 10/1 were reached. The recoveries and precisions were calculated as 100× (final result calculated by the calibration curve/the spiked concentration), and the details are shown in Table 5. The toxin levels of non-detected samples were assumed to be 1/2 LOD.

### 5.5. Food Consumption Data

Food frequency questionnaires were used for collecting food consumption data. Trained investigators were assigned to distribute questionnaires to permanent residents in 11 cities of Zhejiang province between 2015 and 2016, and 6413 residents (live 6 months or more) answered their consumption rates of rice, millet, dried noodles, instant noodles, and maize grains in the recent year. The information of age and body weight was included in the questionnaire for all participants. All participants signed informed consent, and their private information is kept confidential.

### 5.6. The MLs for DON in Human Foods

The MLs of DON were determined according to EU regulation [25] (No 1881/2006, setting maximum levels for certain contaminants in foodstuffs) and Chinese regulation [24] (GB 2761-2017, national standards for food safety, the maximum levels for mycotoxins in foodstuffs). For DON in five foodstuffs, all MLs regulated by EU and China were 750 μg/kg and 1000 μg/kg, respectively. However, the MLs of 3-ADON and 15-ADON were mentioned in neither regulations.

### 5.7. Assessment Methods

The consumption data of each food category by each participant were multiplied by the mean content of each toxin in the food to yield the dietary exposure of each participant relative to that toxin, and the probable daily intake (PDI) of each toxin for each individual was calculated according to the following formula [27]:

(2) $$PDI_{i,j}=\frac{\sum_{\;k=1}^{\;n}C_{i,k}\times L_{k,j}}{BW_{i}}$$

where *PDI_i,j_* is the *PDI* of the contaminant *j* for the individual *i*, *C_i,k_* is the consumption level of the food *k* by the individual *i* (*k* = 1 to N), *L_k,j_* is the mean level of contaminant *j* in the food *k*, and *BWi* is the body weight of the individual *i*. The group *PDI* denotes the sum of *PDI* of DON, 3-ADON, and 15-ADON. The group PMTDI (1 μg/kg bw/day) set by JECFA was used in comparisons with the group *PDI* to assess the risk of the population exposed to DON and its derivatives from grains and grain products. If group *PDI* > group PMTDI, it was considered that the individuals was at a high toxin-exposure level; otherwise, the individual was considered to be at a low toxin-exposure level.

### 5.8. Statistical Analysis

SPSS 15.0 was used to analyze the data, and *p* < 0.05 was set as the level of significance. Measurement data were described by the 50th percentile, the 95th percentile, and mean. Given that the distribution of measurement data was shown as a skewed distribution, pairwise comparisons among groups were performed with a Wilcoxon rank-sum test. Enumeration data were described by rate, and the data were treated with the chi-square test. 

## Figures and Tables

**Table 1:** The prevalence of DON, 3-ADON, and 15-ADON from multiple grain products.

Foodstuffs	Parameter	DON ^a^	3-ADON ^b^	15-ADON ^c^
Rice (*n* = 93)	Percentages (%) ^d^	45.16	11.83	2.15
	No. samples above European Union maximum levels (%)	0/93 (0)	-	-
	No. samples above Chinese maximum levels (%)	0/93 (0)	-	-
	Mean levels (μg/kg)	18.84	5.26	3.94
	P50 levels (μg/kg)	2.50	3.25	3.75
	P95 levels (μg/kg)	77.56	19.90	3.75
	Range (<LOD ^e^-max, μg/kg)	<5.00–131.00	<6.50–29.20	<7.50–13.20
Millet (*n* = 63)	Percentages (%)	7.94	0.00	0.00
	No. samples above European Union maximum levels (%)	0/63 (0)	-	-
	No. samples above Chinese maximum levels (%)	0/63 (0)	-	-
	Mean levels (μg/kg)	14.19	3.75	3.75
	P50 levels (μg/kg)	2.50	3.75	3.75
	P95 levels (μg/kg)	108.41	3.75	3.75
	Range (<LOD-max, μg/kg)	<5.00–266.00	<7.50	<7.50
Dried noodles (*n* = 263)	Percentages (%)	96.96	3.91	0.78
	No. samples above European Union maximum levels (%)	27/263 (10.27)	-	-
	No. samples above Chinese maximum levels (%)	16/263 (6.08)	-	-
	Mean levels (μg/kg)	306.37	5.23	4.72
	P50 levels (μg/kg)	138.00	5.00	5.00
	P95 levels (μg/kg)	960.70	10.00	8.69
	Range (<LOD-max, μg/kg)	<10.00–2697.00	<10.00–56.50	<10.00–10.00
Instant noodles (*n* = 232)	Percentages (%)	95.26	3.45	0.86
	No. samples above European Union maximum levels (%)	18/232 (7.76)	-	-
	No. samples above Chinese maximum levels (%)	12/232 (5.17)	-	-
	Mean levels (μg/kg)	264.72	6.01	4.87
	P50 levels (μg/kg)	136.00	5.00	5.00
	P95 levels (μg/kg)	1223.10	10.00	7.50
	Range (<LOD-max, μg/kg)	<10.00–2416.00	<10.00–120.00	<10.00–11.10
Maize grains (*n* = 62)	Percentages (%)	30.65	1.61	24.19
	No. samples above European Union maximum levels (%)	0/62 (0)	-	-
	No. samples above Chinese maximum levels (%)	0/62 (0)	-	-
	Mean levels (μg/kg)	25.92	3.75	6.61
	P50 levels (μg/kg)	2.50	3.75	3.75
	P95 levels (μg/kg)	111.07	3.75	16.05
	Range (<LOD-max, μg/kg)	<5.00–266.00	<7.50–20.00	<7.50–40.30

^a^ DON, deoxynivalenol. ^b^ 3-ADON, 3-acetyldeoxynivalenol. ^c^ 15-ADON, 15-acetyldeoxynivalenol. ^d^ Percentage of samples in which toxins were detected. ^e^ LOD, limit of detection.

**Table 2:** Daily consumption of grains and grain products of participants per kilogram of body weight.

Foodstuffs	Consumption (g/kg bw/day)											
	Children ^a^ (n = 1494)			Adults ^b^ (n = 4002)			Elderly ^c^ (n = 917)			Total (n = 6413)		
	Mean	P50	P95	Mean	P50	P95	Mean	P50	P95	Mean	P50	P95
Rice	5.80	4.61	12.45	3.86	3.40	7.91	4.22	3.75	8.91	4.36	3.67	9.30
Millet	0.03	0.00	0.04	0.01	0.00	0.02	0.02	0.00	0.03	0.02	0.00	0.03
Dried noodles	0.38	0.17	1.40	0.20	0.09	0.77	0.19	0.07	0.85	0.24	0.10	0.94
Instant noodles	0.07	0.00	0.39	0.03	0.00	0.15	0.01	0.00	0.06	0.04	0.00	0.19
Maize grains	0.17	0.08	0.64	0.10	0.05	0.36	0.10	0.04	0.41	0.12	0.05	0.44

^a^ Children, people aged 0 to 17. ^b^ Adults, people aged 18 to 59. ^c^ Elderly, people aged 60 or older.

**Table 3:** The PDI of DON, 3-ADON, and 15-ADON (μg/kg bw/day).

Foodstuffs	PDI ^a^ (μg/kg bw/day)								
	DON ^b^			3-ADON ^c^			15-ADON ^d^		
	Mean	P50	P95	Mean	P50	P95	Mean	P50	P95
Rice	0.08	0.07	0.18	0.02	0.02	0.05	0.02	0.01	0.04
Millet	<0.01	<0.01	<0.01	<0.01	<0.01	<0.01	<0.01	<0.01	<0.01
Dried noodles	0.07	0.03	0.29	<0.01	<0.01	<0.01	<0.01	<0.01	<0.01
Instant noodles	0.01	<0.01	0.05	<0.01	<0.01	<0.01	<0.01	<0.01	<0.01
Maize grains	<0.01	<0.01	0.01	<0.01	<0.01	<0.01	<0.01	<0.01	<0.01
Total	0.17	0.12	0.44	0.02	0.02	0.05	0.02	0.02	0.04

^a^ PDI, probable daily intake. ^b^ DON, deoxynivalenol. ^c^ 3-ADON, 3-acetyldeoxynivalenol. ^d^ 15-ADON, 15-acetyldeoxynivalenol.

**Table 4:** The group PDI of DON and its derivatives.

Foodstuffs	Group PDI ^a^ (μg/kg bw/day)							
	Children ^b^ (Age = 0–17, n = 1494)		Adults ^c^ (Age = 18–59, n = 4002)		Elderly ^d^ (Age ≥ 60, n = 917)		Total (n = 6413)	
	Mean (% ^e^)	No. Participants at High Exposure Levels ^f^ (% ^g^)	Mean (%)	No. Participants at High Exposure Levels (%)	Mean (%)	No. Participants at High Exposure Levels (%)	Mean (%)	No. Participants at High Exposure Levels (%)
Rice	0.16 (52.43)	5/1494 (0.33)	0.11 (58.60)	2/4002 (0.05)	0.12 (63.56)	0/917 (0)	0.12 (57.13)	7/6413 (0.11)
Millet	<0.01 (0.18)	0/1494 (0)	<0.01 (0.13)	0/4002 (0)	<0.01 (0.22)	0/917 (0)	<0.01 (0.16)	0/6413 (0)
Dried noodles	0.12 (38.77)	11/1494 (0.74)	0.06 (34.92)	4/4002 (0.10)	0.06 (32.40)	2/917 (0.22)	0.08 (35.91)	17/6413 (0.27)
Instant noodles	0.02 (6.67)	0/1494 (0)	0.01 (4.37)	1/4002 (0.02)	<0.01 (1.94)	0/917 (0)	0.01 (4.84)	1/6413 (0.02)
Maize Grains	0.01 (1.96)	0/1494 (0)	<0.01 (1.98)	0/4002 (0)	<0.01 (1.89)	0/917 (0)	<0.01 (1.96)	0/6413 (0)
Total	0.31	32/1494 (2.14)	0.18	11/4002 (0.27)	0.19	3/917 (0.33)	0.21	46/6413 (0.71)

^a^ Group PDI, the sum of probable daily intake of deoxynivalenol, 3-acetyldeoxynivalenol, and 15-acetyldeoxynivalenol. ^b^ Children, people aged 0 to 17. ^c^ Adults, people aged 18 to 59. ^d^ Elderly, people aged 60 or older. ^e^ Contributions of different foodstuffs to toxin exposure. ^f^ Participants whose group PDI > 1 μg/kg bw/day. ^g^ Percentage of participants at high exposure levels.

**Table 5:** Method precision and recovery levels for selected matrices.

Toxins	Added Amount of Toxins (μg/kg)	Rice		Millet		Dried Noodles		Instant Noodles		Maize Grains	
		Recovery (%)	RSD ^d^ (%)	Recovery (%)	RSD (%)	Recovery (%)	RSD (%)	Recovery (%)	RSD (%)	Recovery (%)	RSD (%)
DON ^a^	500	102.92	1.25	102.82	1.83	101.50	4.22	90.17	4.77	103.51	1.52
	1000	100.45	0.57	101.43	0.35	100.08	2.76	93.42	4.26	100.50	0.18
	2000	102.76	1.42	103.50	1.12	87.42	7.41	88.83	2.77	104.83	1.19
3-ADON ^b^	500	98.97	3.61	104.67	2.01	107.01	5.39	106.67	3.46	107.86	4.23
	1000	92.88	3.59	100.98	1.22	110.83	8.10	111.83	1.31	103.33	0.15
	2000	101.70	2.32	105.52	4.75	107.71	8.98	105.83	5.66	106.73	6.99
15-ADON ^c^	500	93.55	2.60	97.36	4.49	97.00	4.22	108.33	11.46	96.12	3.75
	1000	96.89	2.03	94.90	5.86	104.58	9.80	105.67	3.01	93.67	5.48
	2000	95.75	4.79	92.78	3.63	105.42	12.05	107.25	5.42	92.45	2.60

^a^ DON, deoxynivalenol. ^b^ 3-ADON, 3-acetyldeoxynivalenol. ^c^ 15-ADON, 15-acetyldeoxynivalenol. ^d^ RSD, relative standard deviation.

## Data Availability

The data presented in this study are available upon request from the corresponding author due to restrictions, e.g., privacy or ethical.

## Supplementary Materials

The following supporting information can be downloaded at: https://www.mdpi.com/article/10.3390/toxins14090586/s1, Table S1: Representative grains and grains products in individual years.
