# Supplementary material for: Occurrence and Exposure Assessment of Deoxynivalenol and Its Acetylated Derivatives from Grains and Grain Products in Zhejiang Province, China (2017–2020)
Source: Toxins (Basel). 2022 Aug 25;14(9):586. doi: 10.3390/toxins14090586 (PMC9501392; doi:10.3390/toxins14090586)
Supplement: Supplementary file 1 [file toxins-14-00586-s001.zip › toxins-1830066-supplementary.pdf]

toxins

Occurrence and exposure assessment of deoxynivalenol and its acetylated derivatives from grains and grain products in Zhejiang province, China (2017-2020)

Yiming Chen, Ronghua Zhang, Enyu Tong, Pinggu Wu, Jiang Chen, Dong Zhao, Xiaodong Pan, Jikai Wang, Xiaoli Wu, Hexiang Zhang, Xiaojuan Qi, Yinyin Wu, Lei Fang and Biao Zhou

Table S1. Representative grains and grains products in individual years.

Year	Number of food samples				
	Rice	Millet	Dried noodles	Instant noodles	Maize grains
2017	-[a]	-	34	33	-
2018	-	-	39	30	-
2019	93	31	55	38	31
2020	-	32	135	131	31
Total	93	63	263	232	62

[a] Not collected.
